# Optimising Genome‐Wide Detection of Runs of Homozygosity: Impacts of Reference Genome Quality and Sequencing Parameters on Inbreeding Assessment

**DOI:** 10.1111/1755-0998.70084

**Published:** 2025-11-28

**Authors:** Minhui Shi, Haimeng Li, Aaron B. A. Shafer, Tianming Lan

**Affiliations:** ^1^ College of Wildlife and Protected Area Northeast Forestry University Harbin China; ^2^ Heilongjiang Key Laboratory of Complex Traits and Protein Machines in Organisms Harbin China; ^3^ Environmental and Life Sciences Graduate Program Trent University Peterborough Ontario Canada; ^4^ Department of Forensic Science Trent University Peterborough Ontario Canada

**Keywords:** conservation genomics, contig N50, inbreeding, PLINK, sequencing depth

## Abstract

Inbreeding and inbreeding depression pose a critical challenge to the persistence of small and isolated populations, driving the need for precise assessment of genomic metrics. Genome‐wide runs of homozygosity (ROH) have been widely used for evaluating contemporary inbreeding levels and tracing historical events, circumventing the limitations of methods based on pedigree records. However, the reliability of ROH detection is contingent upon the quality of both the reference genome and resequencing data. Here, we employed a simulation‐based approach, generating an inbred population with individuals exhibiting varying inbreeding coefficients and 13 reference genomes with differing levels of contiguity. This framework enabled us to systematically investigate the effects of sequencing depth, read length, reference genome continuity and the phylogenetic divergence of reference genomes on detecting genome‐wide ROH segments. We found that a sequencing depth of ≥ 15× and a reference genome with a contig N50 > 4 Mb enabled discrimination of both the recent and historical inbreeding events, and a reference genome of congeneric subspecies is an optimal choice for ROH detection if a species‐specific reference genome is not available. Furthermore, we performed parameter optimisation for PLINK to enhance ROH detection accuracy under low‐coverage sequencing data and imperfect reference genomes. Our findings established methodological guidance for improving ROH‐based inbreeding assessments, providing critical insights for conservation genomics and breeding programmes where accurate characterisation of genomic homozygosity is paramount.

## 1 Introduction

Endangered species frequently persist in small, isolated populations often as a consequence of habitat fragmentation driven by anthropogenic activities (Haddad et al. 2015; Keyghobadi 2007). In such populations, inbreeding can arise, with subsequent inbreeding depression posing a substantial threat to individual survival and reproductive success (Charlesworth and Willis 2009). Genome‐wide inbreeding signals have emerged as a pivotal metric for assessing the extinction risk of endangered species and small populations (Leutenegger et al. 2003). When inbreeding occurs, the mating of individuals sharing common ancestry leads to the transmission of identical‐by‐descent (IBD) chromosomal segments to their offspring. These IBD segments can be precisely characterised by identifying runs of homozygosity (ROH) across the genome using whole‐genome sequencing (WGS) data (Kardos et al. 2016; Shafer and Kardos 2025).

The longest ROH segments are most likely to emerge in the initial generation of inbreeding and are subsequently fragmented by meiotic recombination over successive generations (Kirin et al. 2010; McQuillan et al. 2008; Thompson 2013). Typically, long ROH segments (> 1 Mb in mammals) indicate recent inbreeding (within 50 generations), while shorter segments often reflect historical inbreeding and demographic events (Curik et al. 2014; Dussex et al. 2021; Khan et al. 2021; Saremi et al. 2019). Additionally, the degree of inbreeding within a population can be quantified by the ratio of the total length of ROH segments to the autosome length (*F*_ROH_, the inbreeding coefficient) (Gazal et al. 2014), which is recognised as a robust genomic measure of inbreeding (Kardos, Åkesson, et al. 2018; Kardos, Nietlisbach, et al. 2018). A higher *F*_ROH_ value signifies more inbreeding in a population, and conversely, a lower value indicates relatively milder inbreeding. Currently, the length distribution and proportion of ROH segments in the genome are widely used to assess both recent and historical inbreeding events in populations (Grossen et al. 2020; Hasselgren et al. 2024; Hoelzel et al. 2024; Kardos et al. 2023; Khan et al. 2021; Kleinman‐Ruiz et al. 2022; Lan et al. 2024; Robinson et al. 2022, 2019; Saremi et al. 2019; Xue et al. 2015). Consequently, achieving precise and comprehensive genome‐wide ROH detection is essential for evaluating individual inbreeding in conservation genomics (Wang et al. 2022; Yang et al. 2023).

For inbred populations, ROH segments often extend over millions of base pairs (Kardos, Åkesson, et al. 2018; Kardos, Nietlisbach, et al. 2018), and the assessment of genome‐wide inbreeding critically depends on both the contiguity of the reference genome and the quality of resequencing data (Lan, Tian, et al. 2025). PLINK is one of the most widely used tools for rule‐based detection of genome‐wide ROH segments, initially developed for SNP array data and later adapted for WGS variants (Purcell et al. 2007). Its robustness has been extensively validated in livestock species, such as cattle and pigs, where ROH‐based inbreeding estimates are routinely applied to manage genetic diversity and mitigate inbreeding depression (Mastrangelo et al. 2018; Schiavo et al. 2020). The principles of ROH detection in livestock research (e.g., parameter thresholds for fragment length and SNP density) are also relevant for WGS‐based research in conservation genomics (Meyermans et al. 2020; Shafer and Kardos 2025). However, the detected ROH segments can exhibit significant variations in length distribution and *F*_ROH_ when different reference genomes are applied, even for the same dataset (Hu et al. 2020; Lan, Tian, et al. 2025; Wang et al. 2022; Wei et al. 2024). Another challenge in accurately evaluating individual inbreeding is that WGS data for endangered species are often obtained from non‐invasive sampling methods, such as faeces, hair or feathers, which typically yield low‐quality data with low sequencing depth making it challenging for many genomics analyses. Furthermore, researchers may have to rely on genomes from closely related species as references to detect ROH segments due to the lack of reference genomes because of severe sampling difficulties, which is not always suitable for identifying ROH segments (Shafer et al. 2016). Therefore, it is necessary in conservation genomics to systematically investigate the impact of these factors on the genome‐wide detection of ROH segments.

With the advent of the long‐read sequencing era, driven by technologies such as Oxford Nanopore Technologies (ONT) and Pacific Biosciences (PacBio) (Clarke et al. 2009; Eid et al. 2009), along with the continuous refinement of assembly algorithms (Amarasinghe et al. 2020), an increasing number of high‐quality reference genomes have been assembled and released (Hotaling et al. 2023), ushering in the era of telomere‐to‐telomere (T2T) genomes (Nurk et al. 2022). Despite the emergence of high‐quality genomes for endangered species, assembling such genomes remains challenging for many species due to sampling difficulties.

As the tiger is among the few endangered species possessing a near T2T (telomere‐to‐telomere) genome at present, it is useful species for evaluating genome‐wide ROH detection. In this study, we simulated an inbred tiger population with individuals exhibiting varying amounts and lengths of ROH segments, based on real tiger genome data, to evaluate the impact of sequencing depth, read length and reference genome continuity on genome‐wide detection of ROH segments. We selected several Felidae species (from subspecies to family level) to investigate the influence of genetic distance between the reference genome and the target population on ROH detection using the PLINK software. Finally, we explored parameter adjustments in PLINK to optimise ROH detection under conditions of poor WGS data and suboptimal reference genomes.

## 2 Materials and Methods

### 2.1 An Overview of the Research Design

**FIGURE 1:**
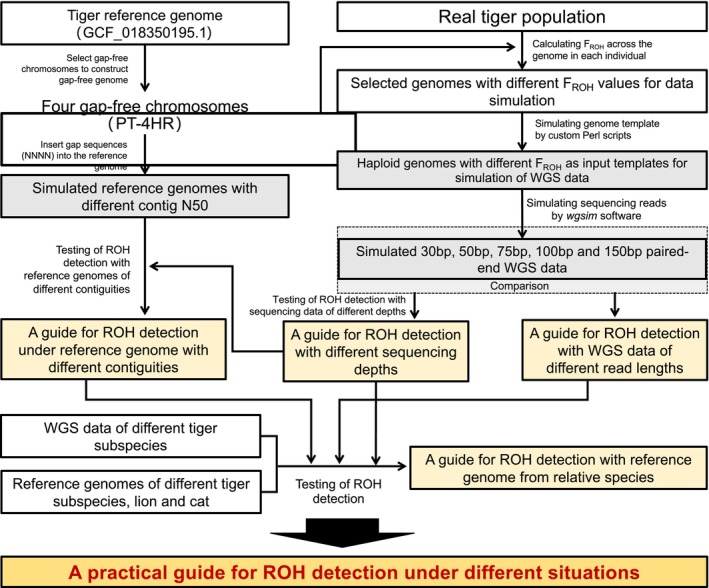
Research design of this study. The grey boxes represent the simulated sequencing data, while the yellow boxes outline the potential guidelines for ROH detection derived from this study.

We evaluated ROH detection from four perspectives (Figure 1). *Group 1*: We examined how sequencing depth affects ROH detection by testing 15 levels of depth: 100×, 60×, 30×, 20×, 15×, 12×, 10×, 8×, 6×, 5×, 4×, 3.5×, 3×, 2.5× and 2×; *Group 2*: We assessed the influence of genome contiguity on ROH detection by including reference genomes with 13 levels of contig N50 ranging from 54.88 Mb to 25.91 kb; *Group 3*: We compared the performance of ROH detection with WGS data of different read lengths (30, 50, 75, 100 and 150 bp); *Group 4*: We tested how the intra‐species and inter‐species genetic distances between the reference genome and the target population influence ROH detection by including reference genome and WGS data from five Felidae species. To ensure the detection of complete ROH segments, we used a gap‐free genome of the Amur tiger as the reference in Groups 1, 2 and 3.

### 2.2 Real Genomic Data Used in This Study

We used the nearly gap‐free tiger genome (GCF_018350195.1) (Bredemeyer et al. 2023) as a reference to evaluate the ROH detection. To ensure the completeness and accuracy of ROH detection, we selected four T2T autosomes (A1: NC_056660.1, B4: NC_056666.1, D4: NC_056672.1, E2: NC_056674.1) from this tiger reference genome to construct a new reference genome (hereafter referred to as PT‐4CHR) for downstream analysis (Bredemeyer et al. 2023; Table S1). The PT‐4CHR was also used for simulating the reference genomes of varying contig N50 (Table S2).

The published WGS data of 13 Amur tigers (Lan, Li, et al. 2025) was used to simulate resequencing data with varying sequencing depths and read lengths. Additionally, WGS data from the South China tiger (CNP0001906, https://db.cngb.org/cnsa/) and Bengal tiger (PRJNA728665 and PRJNA693788, https://www.ncbi.nlm.nih.gov/sra/) were collected to assess the impact of intra‐species genetic distance on ROH detection (Table S3) (Armstrong et al. 2021; Khan et al. 2021; Zhang et al. 2023). Furthermore, reference genomes of the South China tiger (CNA0019679), Amur tiger (CNA0019680), Bengal tiger (GCA_021130815.1), lion (GCF_018350215.1) and domestic cat (GCF_018350175.1) were downloaded to evaluate the influence of inter‐species genetic distance on ROH segment detection (Table S4).

### 2.3 Simulating WGS Data of Different Sequencing Depths

To simulate WGS data of different sequencing depths under different inbreeding levels, we first selected eight individual genomes with different inbreeding levels to be references for the WGS data simulation (Table S3, see Supporting Information). Here we used *F*_ROH_ as the parameter to indicate the inbreeding level, which was calculated by the ratio of the total length of ROH fragments (here we only considered ROH longer than 500 kb identified by PLINK) across the autosomes to the total length of autosomes. The distribution of all ROH segments in each of the eight genomes represents the real state of individual inbreeding in the following data simulation.

**FIGURE 2:**
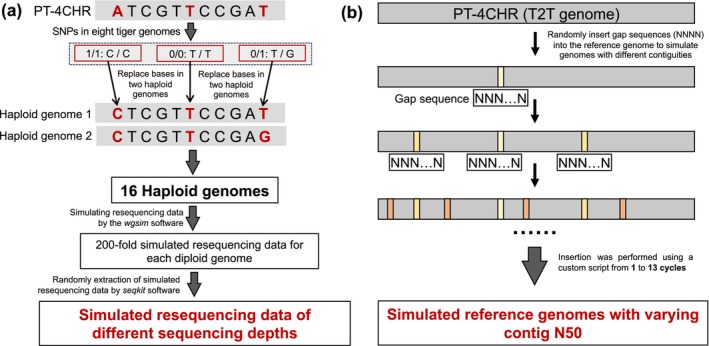
Schematic diagram of the simulation for WGS data and reference genomes. (a) Simulation of WGS data with varying depths. ‘1/1’ denotes a homozygous variant differing from the reference genome; ‘0/0’ indicates a homozygous variant identical to the reference genome; and ‘0/1’ represents a heterozygous variant with one allele differing from the reference genome. (b) Simulation of reference genomes with varying continuity. Up to 13 cycles of N insertions were performed to generate simulated reference genomes with different contig N50 values.

We used the *wgsim* software for data simulation that allowed for the convenient generation of sequencing data of any sequencing depth (up to 100× in this study) based on a single high‐quality tiger genome, with no sequencing error. The WGS data simulation by the *wgsim* software (https://github.com/lh3/wgsim) needs the fasta‐format genome to be the input file. Therefore, for the diploid Amur tiger genome, we first prepared two haploid fasta‐format genomes for each Amur tiger genome by replacing alleles in PT‐4CHR with the corresponding variants in the VCF file, which ensured balanced WGS data simulation for heterozygous sites. For a genotype of ‘1/1’ (homozygous but different from the PT‐4CHR) in the VCF file for an individual, the allele in PT‐4CHR was replaced with the alternative allele in both haploid genomes. For ‘0/1’ genotypes, the replacement was applied to only one of the two haploid genomes. These base replacements were executed using a custom script, available on GitHub: https://github.com/shiminhui/simulation/. This process resulted in 16 fasta‐format haplotype genomes for the eight tiger individuals for the following WGS data simulation (Figure 2a).

We simulated WGS data using the parameters ‘‐1 100 ‐2 100 ‐e 0’, with the above‐constructed fasta‐format haploid genomes as input files. We generated approximately 100 Gb of WGS data (100 bp paired‐end reads) for each haploid genome, equivalent to 200‐fold coverage of the PT‐4CHR. Using the seqkit software (Shen et al. 2016), we randomly selected simulated WGS data to create different data groups with varying sequencing depths, including 100×, 60×, 30×, 20×, 15×, 12×, 10×, 8×, 6×, 5×, 4×, 3.5×, 3×, 2.5×, 2× and 1×. The 1× depth was excluded from the analysis due to the inability to detect ROH segments. To evaluate the effect of read length on ROH detection, we also simulated 30, 50, 75 and 150 bp paired‐end reads for comparison, because 100 and 150 bp are the most commonly used read lengths in current sequencing practice, while shorter read lengths represent ancient DNA or older samples.

### 2.4 Construction of Genomes With Different Contiguities

To generate simulated reference genomes with varying contiguity, we randomly inserted gap sequences (300 bp of ‘N’) into the PT‐4CHR genome, creating a series of simulated reference genomes with a gradient of contig N50 (Figure 2b). The process involved the following steps: (1) randomly inserting 300 consecutive ‘N's into the PT‐4CHR to simulate fragmented reference genomes; (2) evaluating the contig N50 and maximum contig length for each simulated reference genome; and (3) selecting 13 simulated reference genomes with contig N50 values ranging from 25.91 kb to 93.60 Mb for downstream analysis (Table S2). This procedure was performed using a custom script (https://github.com/shiminhui/simulation/).

### 2.5 Testing of ROH Detection With Reference Genomes of Related Species

We collected population WGS data from Bengal tigers (Armstrong et al. 2021; Khan et al. 2021), Amur tigers and South China tigers (Zhang et al. 2023) and mapped these data to high‐quality genomes of five felid species, including the Bengal tiger (PTTI), Amur tiger (PTAL), South China tiger (PTAM), lion (PLEO) and domestic cat (FCAT) (Bredemeyer et al. 2023), to make comparisons between relatives representing different genetic distances to the target species. Here, we assessed the impact of inter‐species and intra‐species genetic distances on the ROH detection and compared by different ROH length categories: ROH > 25 Mb; 25 Mb ≥ ROH > 10 Mb; 10 Mb ≥ ROH > 5 Mb; 5 Mb ≥ ROH > 1 Mb; 1 Mb ≥ ROH > 500 kb.

Before ROH identification, we first compared the PLINK and BCFtools software for detecting ROH (Figure S1). Then, we performed gap‐merging to concatenate the falsely fragmented ROH segments (Ralph and Coop 2013). We found that the difference between average *F*_ROH_ values calculated by PLINK and BCFtools was approximately 5%, which was primarily concentrated in ROH fragments < 500 kb, which may resulted from that the PLINK detection ignores short ROH fragments (Silva et al. 2024). Therefore, after gap‐merging (Ralph and Coop 2013), we retained ROH fragments larger than 500 kb for subsequent analysis (Orkin et al. 2025; Sun et al. 2025). Considering that the PLINK software has been widely used conservation genomics and particularly in tiger populations (Armstrong et al. 2024; Lan, Li, et al. 2025; Zhang et al. 2023), we finally chose PLINK for ROH identification and further analysis.

Furthermore, preliminary analysis suggested that copy number variation (CNV) regions may affect ROH detection, although the observed effects were not statistically significant (*p* > 0.05). Therefore, the CNV was not incorporated into subsequent data simulation and analyses. The basic processes of genome mapping, variant calling, ROH detection using PLINK software (Purcell et al. 2007) and BCFtools (Danecek et al. 2021), gap‐merging, and CNV identification are placed in Supporting Information.

### 2.6 Optimising ROH Detection by Adjusting PLINK Parameters

To improve ROH detection accuracy under conditions of low‐depth WGS data and suboptimal reference genomes, we explored parameter settings in the PLINK software to achieve results closer to the real state. We optimised our parameter settings, based on published studies, to reduce the likelihood of detecting false positive ROHs (Lencz et al. 2007; Purfield et al. 2012). There are two key steps in the detection of ROH by PLINK: (1) In the first step of ROH detection, the command ‘plink ‐‐file filename ‐‐indep‐pairwise n1 n2 n3’, which calculates LD between SNP pairs within a *n1* kb window, advancing *n2* SNP per iteration. If the *r* ^2^ value of any SNP pair in the window exceeds *n3*, one SNP is marked as redundant and removed; (2) The filtered SNP set was then passed to the second step for ROH detection with the parameters ‘‐‐homozyg ‐‐homozyg‐window‐snp n4 ‐‐homozyg‐density n5 ‐‐homozyg‐kb n6 ‐‐homozyg‐snp n7’. This step employs a sliding window of *n4* SNPs to scan the genome, allows a maximum of *n5* SNPs per Mb to control SNP density, and identifies regions longer than *n6* kb with at least *n7* SNPs as ROH. To refine the ROH detection, we adjusted several parameters in both of the two steps, including varying the window size of ‘‐‐indep‐pairwise’ (*n1*) from 1 Mb to 5 kb, the *r* ^2^ threshold (*n3*) from 0.01 to 0.99 (Meyermans et al. 2020), the ‘‐‐homozyg‐window‐snp’ parameter (*n4*) from 100 SNPs to 5 SNPs and the ‘‐‐homozyg‐snp’ parameter (*n7*) from 50 SNPs to 300 SNPs.

## 3 Results

### 3.1 Simulated WGS Data and Reference Genomes

In total, we simulated eight groups of WGS data and 13 reference genomes to evaluate ROH detection under different conditions (Table 1). The eight groups of WGS data represented an inbred population consisting of individuals exhibiting eight different inbreeding levels (*F*_ROH_ = 5.66%, 12.37%, 17.17%, 22.10%, 28.41%, 35.43%, 38.54%, 47.73%) (Tables S3 and S5), and each group of WGS data included 15 different levels of sequencing depth ranging from 2× to 100×. The length of these simulated paired‐end reads included 30, 50, 75, 100 and 150 bp, which could represent a diverse read length panel in WGS sequencing, even for ancient DNA sequencing. For the 13 reference genomes, we used the PT‐4CHR (Contig N50: 93.60 Mb, Max Contig: 155.91 Mb) to represent the perfect reference genome. By randomly inserting gaps (Ns) into the PT‐4CHR, we finally generated 13 additional reference genomes with contig N50 ranging from 50.88 Mb to 25.91 kb and maximum contig lengths ranging from 130.94 Mb to 800.19 kb (Table S2).

**TABLE 1:** Genomic data used for testing of ROH detection under different conditions.

Testing groups	Reference genome and contig N50	WGS data	Sequencing depth (×)	Read length (bp)
Group 1	PT‐4CHR (Contig N50 = 93.60 Mb)	Eight groups of simulated WGS data with varying inbreeding levels (*n* = 8)	15 levels of sequencing depths (see Section 2.1)	100
Group 2	13 levels of contig N50 ranging from 54.88 Mb to 25.91 kb (see Table S2)	Eight groups of simulated WGS data with varying inbreeding levels (*n* = 8)	20	100
Group 3	13 levels of contig N50 ranging from 54.88 Mb to 25.91 kb (see Table S2)	Eight groups of simulated WGS data with varying inbreeding levels (*n* = 8)	20	30, 50, 75, 100 and 150
Group 4	South China tiger: CNA0019679, Contig N50 = 9.47 Mb; Amur tiger: CNA0019680, Contig N50 = 9.52 Mb; Bengal tiger: GCA_021130815.1, Contig N50 = 4.4 Mb; Lion: GCF_018350215.1, Contig N50 = 77.8 Mb; Domestic cat: GCF_018350175.1, Contig N50 = 90.7 Mb	South China tiger (*n* = 15); Amur tiger (*n* = 13); Bengal tiger (*n* = 12)	South China tiger: 19.47 ± 2.19; Amur tiger: 20.59 ± 2.52; Bengal tiger: 20.74 ± 4.26	100

*Note:* Groups 1–4 see Section 2.1.

### 3.2 Optimal Sequencing Depth for ROH Detection

**FIGURE 3:**
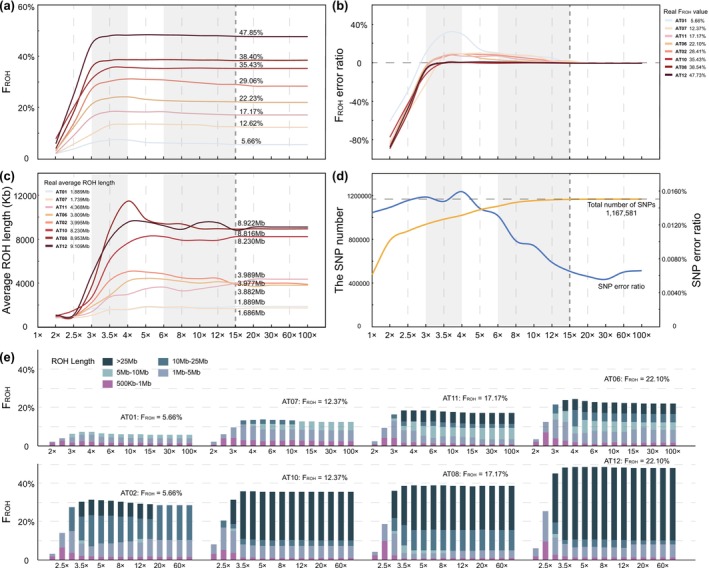
The impact of sequencing depth on genome‐wide ROH detection. (a) *F*_ROH_ as a function of sequencing depth. The legend on the right indicates the real *F*_ROH_ values of the simulated individual genomes. (b) The *F*_ROH_ error ratio at different sequencing depths. (c) The average length of detectable ROH segments at varying sequencing depths. (d) The number of detected SNPs (yellow) and the SNP error ratio (blue, the number of erroneously called SNPs/the total number of SNPs) at different sequencing depths. The dashed line represents the actual number of SNPs. (e) Percent of genome in runs of different size categories for the test individuals when sequencing coverage is set to different depths.

The detectable ROH segments in the genome approach the real (i.e., known) state only when the individual sequencing depth reaches or exceeds 15× (Figure 3a). We refere to the differnce as the *F*_ROH_ error ratio: (detected *F*_ROH_ − real *F*_ROH_)/real *F*_ROH_. At the sequencing depth of 15×, the *F*_ROH_ error ratio was only 0.66% ± 1.01%, and then gradually decreased to 0.02% ± 0.07% at 100× (Figure 3b). Interestingly, the detectable *F*_ROH_ exhibited a non‐linear relationship with the sequencing depth below 15× (Figure 3a, Table S6). At 4×, the error ratio was higher for less inbred individuals (e.g., AT01: *F*_ROH_ = 5.66%) when compared to highly inbred ones (AT12: *F*_ROH_ = 47.73%) (Figure 3b, Table S6). For accurate *F*_ROH_ assessment, we recommend a minimum sequencing depth of 15×. Lower depths may underestimate (< 3×) or overestimate (3× to 15×) the *F*_ROH_, and this bias was more evident for individuals with *F*_ROH_ < 30%. However, for *F*_ROH_ > 30%, depths above 3× could reliably reflect the real inbreeding levels.

We then analysed the length of detectable ROH segments under different sequencing depths (Figure 3c). The average length of detectable ROH increased rapidly from 2× to 4×, slightly declined from 4× to 6×, and then steadily decreased from 6× and approaching the real length at 15× or higher sequencing depths. This pattern may reflect the increasing accuracy of SNP detection with increasing sequencing depth (Figure 3d). For studies of historical inbreeding (requiring precise detection of ROH length), we recommend a minimum sequencing depth of 15×. For samples with limited available DNA (e.g., faecal or ancient specimens), we recommend maintaining no < 5× depth as a critical threshold, though the *F*_ROH_ may be overestimated, particularly for low‐inbreeding populations.

For both the *F*_ROH_ and the average length of detectable ROH segments, we observed an unexpected peak value at the sequencing depth of 4× (Figure 3a,c). This non‐linear pattern may reflect a trajectory of SNP error ratio as a function of sequencing depth (Figure 3d): at sequencing depths lower than 3×, insufficient detected SNPs could lead to the underestimation of ROHs (Figure 3e), while the overestimation of ROH was gradually eliminated under the increasing sequencing depth.

Given that 5× is often recommended for ROH detection for low‐quality samples (Femerling et al. 2023), we therefore optimised PLINK parameters for ROH detection under this sequencing depth. Additionally, we referred to the method of Lencz et al. (2007) to further refine our parameters for accurate ROH detection. Reducing the parameter ‘‐‐homozyg‐window‐snp’ from 100 to 20 increased both *F*_ROH_ and average ROH length. For *F*_ROH_, setting this parameter to 80 yielded *F*_ROH_ values close to the real value in most individuals, while 70 was more suitable for highly inbred individuals (*F*_ROH_ ≈ 50%). For ROH length, however, we recommend ‘‐‐homozyg‐window‐snp 20’, despite the potential underestimation (Figure S4, Table S7). Furthermore, we found that the parameters of ‘‐‐homozyg‐snp 150’ and ‘‐‐indep‐pairwise 50 1 0.8’ could also improve the accuracy of ROH detection under low sequencing depth. In summary, for low‐depth WGS data, we recommend ‘‐‐indep‐pairwise 50 1 0.8; ‐‐homozyg‐window‐snp 80; ‐‐homozyg‐snp 150’. For individuals with *F*_ROH_ > 50%, reducing ‘‐‐homozyg‐window‐snp’ to 70 appears more appropriate.

### 3.3 The Impact of Read Lengths on ROH Detection

**FIGURE 4:**
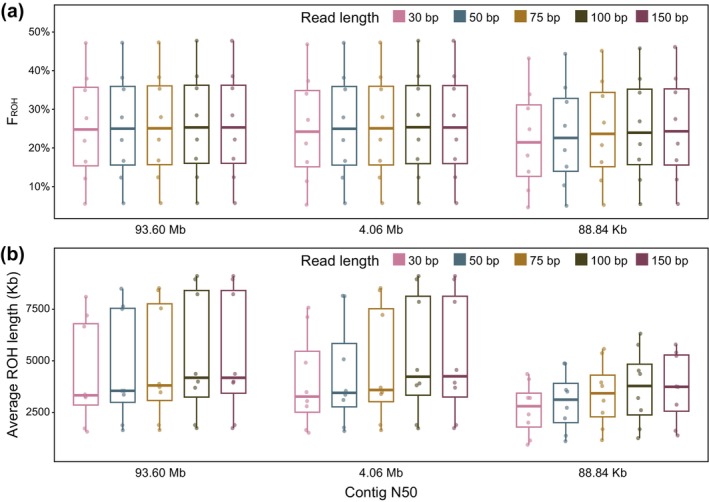
The impact of read length on genome‐wide ROH detection. No significant differences were observed among 30, 50, 75, 100 and 150 bp sequencing read lengths in terms of *F*_ROH_ (a) or average ROH length (b).

Using the same set of reference genomes, we separately calculated *F*_ROH_ and average ROH length with both 100 and 150 bp simulated sequencing reads and compared the results. No significant differences in ROH detection were observed between the two read lengths (Figure 4, Figure S4, Tables S8 and S9). In light of some particular cases (e.g., ancient DNA research), shorter read lengths occur in whole‐genome resequencing. In this study, we also investigated the impact of shorter read lengths (30, 50 and 75 bp) on ROH detection to simulate sequencing of low‐quality samples in ancient DNA research like the situation. We found that although ROH detection performed better as read length increases, the read length did not significantly affect ROH detection (Figure 4, Table S10). However, we cannot conclude that the ROHs are well detected in ancient DNA research with sequencing data of short‐read lengths, because (1) the sequencing depth in ancient DNA research is usually very low; (2) there is DNA damage in ancient DNA sequences; (3) many reads with length shorter than 30 bp also existed in ancient materials.

### 3.4 Impact of the Reference Genome Contiguity on ROH Detection

**FIGURE 5:**
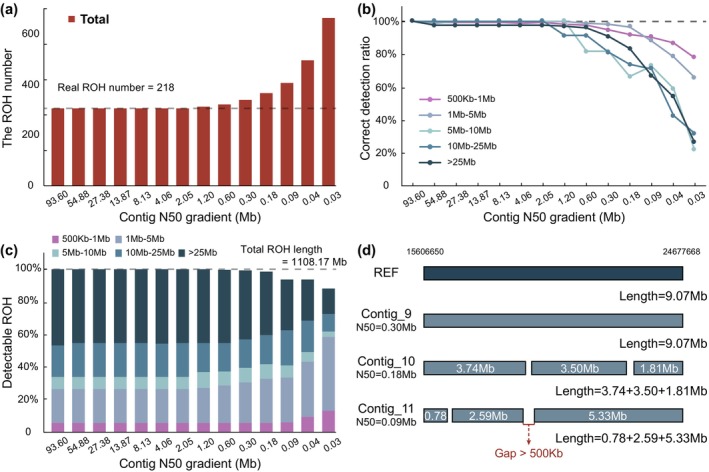
The impact of reference genome continuity on ROH detection. (a) Total number of ROH segments detected using reference genomes of varying continuities. The actual number of ROH segments in the simulated population is 462. (b) Correct detection ratio of ROH segments across different lengths under reference genomes of varying continuities. (c) Detectable ROH segments of different lengths under reference genomes of varying continuities. The total ROH length in the simulated population is 1148.58 Mb. (d) Illustration of how a fragmented reference genome introduces erroneous or omitted SNP sites, leading to the breakup of longer ROH segments.

We found that the total number of detectable ROH segments remained stable as the reference genome's contig N50 decreased from 93.60 to 0.6 Mb. However, a significant increase in detectable ROH segments occurred when the contig N50 dropped to below 0.6 Mb (Figure 5a), indicating that larger ROH segments were fragmented during the detection as genome continuity declined.

Our study identified two critical thresholds for ROH detection: the contig N50 of 2 and 0.6 Mb in the reference genome. When the contig N50 reached 2 Mb, both *F*_ROH_ and the average length of ROH segments could be accurately assessed (Figure 5a, Table S8). As the contig N50 decreased, the average length of detectable ROH segments declined (Figure 5b, Figure S6). However, *F*_ROH_ was more tolerant to reference genome fragmentation and could still be accurately evaluated even when the contig N50 dropped to 0.6 Mb (Figure 5c, Table S8). These findings suggest that reference genomes with a contig N50 > 0.6 Mb are sufficient for assessing overall individual inbreeding, but a more complete genome (contig N50 > 2 Mb) is necessary to fully trace inbreeding history.

Although we used gap‐merging strategy to correct falsely fragmented ROH, some cases were still difficult to be corrected. For example, a 9.07 Mb ROH in chromosome B4 of individual AT06 could be completely detected based on a reference genome with a contig N50 longer than 0.3 Mb. However, this ROH fragment was broken into several small fragments by the low‐quality reference genome (Figure 5d). Based on current gap‐merging rules, these fragments could not be concatenated by the gap‐merging process because they were either shorter than 4 Mb or the gaps between them were longer than 500 kb.

Although current reference genomes often achieve contig N50 values > 2 Mb (Amarasinghe et al. 2020), many databases are still filled with short‐read assemblies with contig N50 < 100 kb (Hotaling et al. 2023). To mitigate the underestimation of inbreeding with fragmented genomes, we adjusted the ‘‐‐homozyg‐window‐snp’ parameter to reduce the number of SNPs in a window, as some ROH segments may span multiple contigs, leading to false negatives (Figure S6). Lowering this parameter increased detectable ROH segments but did not significantly affect their average length. For reference genomes with contig N50 < 100 kb, we recommend setting ‘‐‐homozyg‐window‐snp’ to 10–15, while keeping other parameters at their optimal values (Table S11).

### 3.5 Impact of Genetic Distance on ROH Detection

**FIGURE 6:**
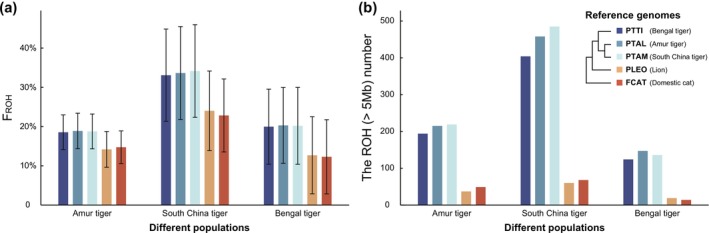
ROH detection in populations of three tiger subspecies (Amur tiger, South China tiger, Bengal tiger) using reference genomes from five felid species (Amur tiger, South China tiger, Bengal tiger, lion and cat). (a) Comparison of *F*_ROH_ detection with reference genomes from different relative species. (b) Comparison of the number of ROH fragments larger than 5 Mb detected with reference genomes from different relative species. The genetic relationship among Felidae species is from the findings of Sun et al. (2023), with branch lengths omitted.

In real‐world scenarios, genomic analysis often relies on reference genomes from closely related species or subspecies due to sampling challenges in endangered species (Dehasque et al. 2024; Van der Valk et al. 2021). Using high‐quality reference genomes (contig N50 > 4 Mb) from five felid species and high‐depth WGS data (> 15×) from three tiger populations, we aimed to establish a strategy for selecting an appropriate reference genome to assess individual inbreeding. *F*_ROH_ in tiger genomes calculated using reference genomes of three tiger subspecies was highly consistent (Figure 6a). In contrast, *F*_ROH_ of tiger genomes calculated by using lion and domestic cat genomes as the reference was significantly underestimated (Table S12).

Analysis of the length distribution of detectable ROH segments revealed a notable disparity at the 5 Mb threshold (Table S13, Figure S7). For ROH segments < 5 Mb, the number of detectable ROHs increased with genetic distance (Figure S7), which is more evident in the lion and cat groups. In contrast, the number of ROH segments > 5 Mb detected using the three tiger reference genomes was significantly higher than those detected using the lion and cat genomes. Additionally, for ROH segments longer than 5 Mb, we observed that the number of ROHs detected in the Amur tiger and South China tiger populations was lower when using the Bengal tiger genome as a reference compared to the Amur tiger and South China tiger genomes (Figure 6b). This suggests that the ROH detection could also be influenced by the genetic distance even at the subspecies level.

We further adjusted PLINK parameters to optimise ROH detection when employing a reference genome from a closely related species. By using the lion genome to detect ROHs in the Bengal tiger population, we found that a smaller value for ‘‐‐indep‐pairwise’ and ‘‐‐homozyg‐window‐snp’ increased *F*_ROH_, while the average ROH length was positively correlated with ‘‐‐indep‐pairwise’ and negatively correlated with ‘‐‐homozyg‐window‐snp’ (Figure S8, Table S14). We do not recommend specific parameters due to taxon‐specific variability, but for big cats, we suggest lowering ‘‐‐indep‐pairwise’ and ‘‐‐homozyg‐window‐snp’ for *F*_ROH_ evaluation and enhancing ‘‐‐indep‐pairwise’ for assessment of ROH length. It is important to note that ROH detected through parameter adjustments may not accurately reflect the true ROH distribution in the genome.

## 4 Discussion

Inbreeding is a critical factor contributing to the extinction of animals in small and isolated populations (Charlesworth and Willis 2009). Accurate assessment of inbreeding is essential for biodiversity conservation, especially in the context of the ongoing sixth mass extinction (Barnosky et al. 2011; Ceballos et al. 2015; Pimm et al. 2014). For conservation genetic studies of many endangered species challenges from sampling difficulties and the lack of high‐quality samples remain prevalent (Cui et al. 2024; Orlando et al. 2021; Yuan et al. 2021). These challenges influence the accurate evaluation of inbreeding through ROH analysis, often due to low‐coverage WGS data and suboptimal reference genomes. For instance, the inbreeding level of an inbred Chinese pangolin population was initially assessed using *F*_ROH_ based on a short‐read assembled reference genome (Hu et al. 2020). Subsequent re‐evaluations of the same population using different reference genomes yielded significantly different *F*_ROH_ results: specifically, higher *F*_ROH_ could be detected by using the long‐read assembled genome than that detected by the reference genome assembled with short‐read sequencing data, particularly for ROHs longer than 1 Mb (Lan, Tian, et al. 2025; Wang et al. 2022; Wei et al. 2024). Current discussions on ROH detection include comparisons of different methods for inbreeding evaluation (Lavanchy and Goudet 2023) and the influence of individual nucleotide diversity or genome recombination rates on ROH distribution (Ceballos et al. 2018). While some studies have explored the impacts of reference genome quality and sequencing depth on genome‐wide ROH detection (Kardos and Waples 2024; Lan, Tian, et al. 2025; Shafer et al. 2016), a practical and widely applicable guideline for ROH detection remains lacking, particularly for widely used program PLINK.

In this study, we first investigated the optimal sequencing depth of WGS data for evaluating individual inbreeding through ROH detection. We identified 15× as a threshold depth for accurate and complete detection of ROH segments across the genome (Figure 3), a finding consistent with previous research (Kardos and Waples 2024). This is primarily because SNPs used for ROH detection can be accurately and fully identified at this depth. Interestingly, the relationship between detectable ROH and sequencing depth is not linear. *F*_ROH_ rapidly increases to a peak at 4× depth, then gradually decreases and converges to the true value. Consequently, *F*_ROH_ at 5× depth closely approximates the real *F*_ROH_, making it a viable alternative for low‐quality samples (Femerling et al. 2023). However, it is important to note that this depth is insufficient for tracing historical inbreeding events, as it results in numerous inaccurately detected ROH segments and an average ROH length deviates from the true value (Figure 3c). Notably, recent advances in human genomics have demonstrated that a high‐quality reference panel could facilitate the accurate ROH detection at much lower sequencing depth (0.4–0.5×) through haplotype‐based approaches (1000 Genomes Project Consortium 2015; Bai et al. 2018). Although such methods currently require extensive population‐specific reference genetic resources (Ringbauer et al. 2021; Sousa da Mota et al. 2023), they indicate potential future directions for conservation genomics if similar reference datasets could be available for endangered species. Regarding the selection of reference genome contiguity, we found that a contig N50 of 2 Mb is adequate for evaluating both current and historical inbreeding events. As the contig N50 decreases, ROH segments become increasingly fragmented, yet *F*_ROH_ remains accurate if the contig N50 exceeds 0.6 Mb, a benchmark readily achieved with long‐read sequencing technology (Amarasinghe et al. 2020; Hotaling et al. 2023). However, as genome continuity further declines, we observed significant fragmentation of ROH segments longer than 5 Mb and a corresponding increase in the number of detected ROHs. Furthermore, our results indicated that the ROH detection was not affected by sequencing read length. In ancient DNA research, however, careful consideration must be given to both limited read lengths and sequencing depth, due to the low sequencing quality in ancient materials.

Another common challenge in ROH detection for endangered species is the selection of a reference genome from a related species, such as using the genome of an extant elephant to evaluate inbreeding in woolly mammoth populations (Dehasque et al. 2024). In this study, we confirmed that the closer the genetic relationship between the reference genome and the target population, the more accurate the ROH detection (Figure 6). Using documented Felidae divergence times—12.6 million years (Ma) between domestic cats and pantherine lineages, and 4.58 Ma between lions and tigers (Figueiró et al. 2017), we observed that significantly more ROH segments shorter than 5 Mb were detected in tiger genomes when using lion and cat reference genomes compared to tiger reference genomes, indicating that long ROH segments (> 5 Mb) were fragmented when lion and cat genomes were used as references for ROH detection in tigers (Table S13). Based on the synteny relationships among Felidae genomes (Bredemeyer et al. 2023), structural variants (SVs) between closely related species result in mosaic‐like collinearity, making the genome of a closely related species function similarly to a fragmented reference genome for ROH analysis in the target species. However, the genome sequences and structures among tiger subspecies are highly similar, leading to comparable ROH detection results across tiger subspecies reference genomes. Notably, the accuracy of ROH detection in Amur tigers was slightly higher when using the South China tiger reference genome compared to the Bengal tiger reference genome. This can be attributed to the closer genetic relationship between Amur tigers and South China tigers, whereas Bengal tigers diverged from other continental tigers approximately 52,920 years ago, much earlier than the divergence between Amur tigers and South China tigers (Sun et al. 2023; Zhang et al. 2023). Although we concluded that a reference genome from subspecies instead of the species‐specific reference genome is an optimal choice for ROH detection, it is difficult to conclude that this recommendation could satisfy all animal taxa because the genetic divergence among species may be very different. Nonetheless, the feline model provides a reference for the ROH detection of endangered animals whose genomes are unavailable.

## 5 Conclusions

**FIGURE 7:**
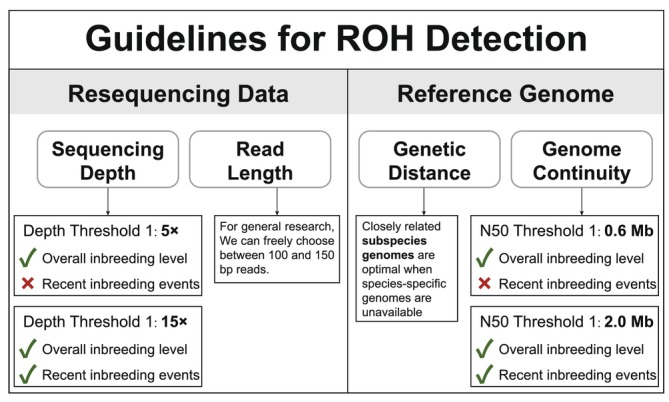
A practical guide to genome‐wide ROH detection in conservation genomics.

We have developed practical guidelines for the detection of ROH under diverse scenarios (Figure 7). (1) For accurate and complete genome‐wide ROH detection, a sequencing depth exceeding 15× is generally recommended. The evaluation of *F*_ROH_ demonstrates greater tolerance to variations in sequencing depth compared to the assessment of average ROH length. Specifically, a sequencing depth of 5× is sufficient for precise *F*_ROH_ calculation, and this can be further reduced to 3× for individuals exhibiting high inbreeding levels (e.g., *F*_ROH_ > 30%). (2) Read length appears to be a less critical factor in ROH detection, suggesting minimal impact on the accuracy of the analysis. (3) A reference genome with a contig N50 > 0.6 Mb is adequate for *F*_ROH_ estimation, while a more complete genome (contig N50 > 2 Mb) is necessary to trace historical inbreeding events. (4) When a species‐specific reference genome is unavailable, utilising a reference genome from a closely related subspecies is an optimal approach for ROH detection, outperforming the use of reference genomes from different species, especially those outside the same genus. (5) The parameters ‘‐‐homozyg‐window‐snp’, ‘‐‐homozyg‐snp’ and ‘‐‐indep‐pairwise’ can enhance ROH detection with low‐depth WGS data. We recommend adjusting the pairwise *r* ^2^ threshold of ‘‐‐indep‐pairwise’ to 0.8, increasing ‘‐‐homozyg‐window‐snp’ to 70–80, and setting ‘‐‐homozyg‐snp’ to approximately 150 to improve *F*_ROH_ accuracy when using suboptimal reference genomes. (6) For ROH detection using reference genomes across different species, we do not recommend specific PLINK parameters as suitable options, as their performance may heavily depend on the taxonomic group, even though parameter adjustments could enhance detection accuracy.

## Author Contributions

T.L. designed the study and supervised the project. M.S. and H.L. carried out the bioinformatic analysis. T.L., M.S. and H.L. wrote the manuscript. A.B.A.S. and T.L. extensively revised the manuscript.

## Funding

This work was supported by the Guidance Category Project of the Key Research and Development Plan in Heilongjiang Province (GZ2024010), China Postdoctoral Science Foundation (2025M771914), National Natural Science Foundation of China (32570587) and the Fundamental Research Funds for the Central Universities (2572025JT07).

## Conflicts of Interest

The authors declare no conflicts of interest.

## Supporting information


**Appendix S1:** men70084‐sup‐0001‐AppendixS1.docx.

**Appendix S2:** men70084‐sup‐0002‐AppendixS2.xlsx.

## Data Availability

The reference genomes involved in this study are all from public databases (NCBI, https://www.ncbi.nlm.nih.gov/, and CNGBdb, https://db.cngb.org/cnsa/), including GCF_018350195.1, GCA_021130815.1, GCF_018350215.1, GCF_018350175.1, CNA0019679, CNA0019680. All resequencing data used are also from the above two public databases: Amur tiger population and South China tiger population: CNP0001906 (CNGBdb), Bengal tiger population: PRJNA728665 (NCBI) and PRJNA693788 (NCBI). The custom codes are available on GitHub (https://github.com/shiminhui/simulation/). The common analysis pipelines used in data processing were all executed according to the manual and protocols of the corresponding bioinformatics software. The specific versions of software have been described in Section 2. Any additional information in this study is available from the lead contact upon request.
