# Supplementary material for: Optimising Genome‐Wide Detection of Runs of Homozygosity: Impacts of Reference Genome Quality and Sequencing Parameters on Inbreeding Assessment
Source: Mol Ecol Resour. 2025 Nov 28;26(1):e70084. doi: 10.1111/1755-0998.70084 (PMC12661482; doi:10.1111/1755-0998.70084)
Supplement: Supplementary file 1 — Appendix S1: men70084‐sup‐0001‐AppendixS1.docx. [file MEN-26-e70084-s002.docx]

**Supplementary Materials**

**Basic workflow of ROH detection**

To simulate genomes with different inbreeding levels (F_ROH_), we first detected ROH segments in 30 real Amur tiger genomes (Lan et al., 2024; Zhang et al., 2023) to selected suitable individual genome for simulation.

We initially aligned WGS data of the 30 Amur tigers to the reference genome (GCF_018350195.1) using BWA (v0.7.17) *mem* algorithm with default parameters. The mapped sequences were then classified and converted into a BAM file using the *view* command in SAMtools (v1.7) (Danecek et al., 2021). The *SortMarkDup* pipeline of ZBOLT (MegaBOLT v2.3.0.0, https://en.mgi-tech.com/products/software_info/6) was employed for sorting and deduplicating the BAM files. Raw variants for each individual were called using the HaplotypeCaller algorithm in GATK (v4.1.2.0) (DePristo et al., 2011), generating gVCF (Genomic Variant Call Format) files. These gVCF files were subsequently merged into a single combined gVCF file using the CombineGVCFs tool in GATK (v4.1.2.0) (DePristo et al., 2011). Joint variant calling was performed using *GenotypeGVCFs* in GATK to produce a population-level VCF file. SNPs were extracted from all variants using GATK (v4.1.2.0) with the parameter “*SelectVariants --select-type-to-include SNP*”, followed by hard filtering with the criteria “*QD < 2.0 || MQ < 40.0 || FS > 60.0 || MQRankSum < -12.5 || ReadPosRankSum < -8.0*”. The SNP set was further refined using VCFtools (v0.1.16) (Danecek et al., 2011) to remove all non-bi-allelic SNPs and those with a missing rate exceeding 20%.

To detect ROH fragments, the multi-step filtered VCF file was converted into PLINK file format using the PLINK software (v1.07) (Purcell et al., 2007), and the SNP set was pruned using the “--indep-pairwise” option with the window size of 50 Kb and a pairwise r^2^ threshold value of 0.9. ROH segments were subsequently detected using PLINK software (v1.07) with the widely applied parameters “--homozyg --homozyg-window-snp 20 --homozyg-density 50 --homozyg-kb 10” (Sun et al., 2025; Yuan et al., 2024; Yuan et al., 2023; Zhang et al., 2023).

Using the same VCF file described above, we also used the BCFtools (v1.21) (Danecek et al, 2021) to identify ROH segments. The filtered VCF file was first converted to BCF format and indexed. The BCFtools roh module was then used for ROH detection with the default parameters *“bcftools roh -G30 --AF-dflt 0.4 vcf.gz -o out_0.4.txt --output-type r”*, and retained fragments with average fwd-bwd phred score >70 (Orkin et al., 2025).

The preliminary ROH results detected using PLINK and BCFtools were further processed by a gap-merging method to concatenate wrongly interrupted long ROH segments. In this study, we prepared a custom Perl script to carry out the gap-merging process by merging ROH fragments with anchor lengths greater than 4 Mb and with gaps between them that were less than 0.5 Mb (Ralph & Coop, 2013). After gap-merging, we further remove ROH fragments shorter than 500Kb for the subsequent analysis (Goli et al., 2024; Orkin et al., 2025). The F_ROH_ was calculated by the following format: the total length of ROH segments (>500Kb) in PT-4CHR / the total length of the PT-4CHR.

**Basic workflow of CNV detection**

CNVnator software (v0.4.1) (Abyzov et al., 2011) was used for copy number variation (CNV) detection. First, the labeled BAM files were converted to ROOT format. A read depth distribution histogram was constructed based on the reference genome at a resolution of 1000 bins and statistical analysis was performed. Candidate CNV regions were identified by genomic partitioning at the same resolution. Finally, the detection results, including deletion and duplication variants, were output to sample-specific files.

**Reference**

Abyzov, A., Urban, A. E., Snyder, M., & Gerstein, M. (2011). CNVnator: an approach to discover, genotype, and characterize typical and atypical CNVs from family and population genome sequencing. *Genome Research, 21*(6), 974-984.

Danecek, P., Auton, A., Abecasis, G., Albers, C. A., Banks, E., DePristo, M. A., et al. (2011). The variant call format and VCFtools. *Bioinformatics, 27*(15), 2156-2158.

Danecek, P., Bonfield, J. K., Liddle, J., Marshall, J., Ohan, V., Pollard, M. O., et al. (2021). Twelve years of SAMtools and BCFtools. *Gigascience, 10*(2), giab008.

DePristo, M. A., Banks, E., Poplin, R., Garimella, K. V., Maguire, J. R., Hartl, C., et al. (2011). A framework for variation discovery and genotyping using next-generation DNA sequencing data. *Nature genetics, 43*(5), 491-498.

Goli, R. C., Mahar, K., Manohar, P. S., Chishi, K. G., Prabhu, I. G., Choudhary, S., et al. (2024). Insights from homozygous signatures of cervus nippon revealed genetic architecture for components of fitness. *Mammalian Genome, 35*(4), 657-672.

Lan, T., Li, H., Liu, B., Shi, M., Tian, Y., Sahu, S. K., et al. (2024). Revealing extensive inbreeding and less-efficient purging of deleterious mutations in wild Amur tigers in China. *Journal of Genetics and Genomics*.

Orkin, J. D., Kuderna, L. F., Hermosilla-Albala, N., Fontsere, C., Aylward, M. L., Janiak, M. C., et al. (2025). Ecological and anthropogenic effects on the genomic diversity of lemurs in Madagascar. *Nature ecology & evolution, 9*(1), 42-56.

Purcell, S., Neale, B., Todd-Brown, K., Thomas, L., Ferreira, M. A., Bender, D., et al. (2007). PLINK: a tool set for whole-genome association and population-based linkage analyses. *The American Journal of Human Genetics, 81*(3), 559-575.

Ralph, P., & Coop, G. (2013). The geography of recent genetic ancestry across Europe. *PLoS biology, 11*(5), e1001555.

Sun, L., Yuan, C., Guo, T., Bai, Y., Lu, Z., & Liu, J. (2025). The accumulation of harmful genes within the ROH hotspot regions of the Tibetan sheep genome does not lead to genetic load. *BMC genomics, 26*(1), 1-13.

Yuan, J., Kitchener, A. C., Lackey, L. B., Sun, T., Jiangzuo, Q., Tuohetahong, Y., et al. (2024). The genome of the black-footed cat: Revealing a rich natural history and urgent conservation priorities for small felids. *Proceedings of the National Academy of Sciences, 121*(2).

Yuan, J., Wang, G., Zhao, L., Kitchener, A. C., Sun, T., Chen, W., et al. (2023). How genomic insights into the evolutionary history of clouded leopards inform their conservation. *Science Advances, 9*(40), eadh9143.

Zhang, L., Lan, T., Lin, C., Fu, W., Yuan, Y., Lin, K., et al. (2023). Chromosome‐scale genomes reveal genomic consequences of inbreeding in the South China tiger: A comparative study with the Amur tiger. *Molecular Ecology Resources, 23*(2), 330-347.

**Supplementary Figures**


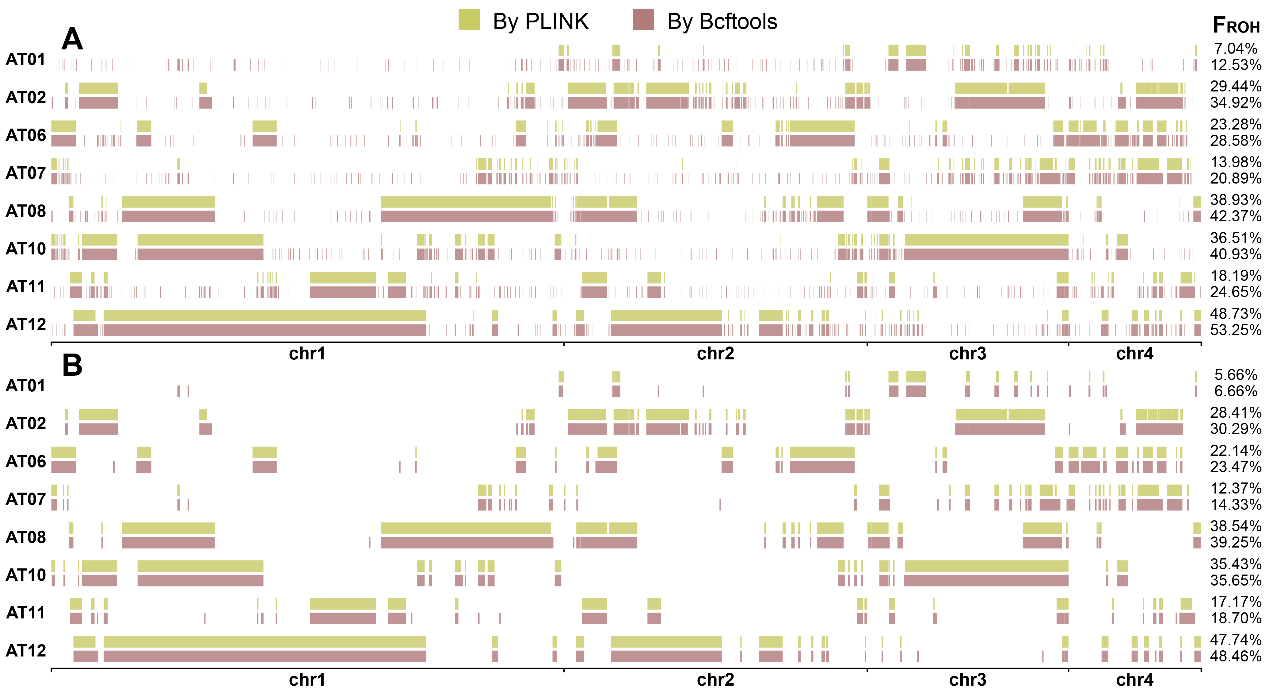


Figure S1. The distribution patterns of ROH detection results of PLINK and Bcftools software on the genome. (A) The original ROH results were merged. (B) The merged ROH was filtered to remove fragments less than 500Kb.


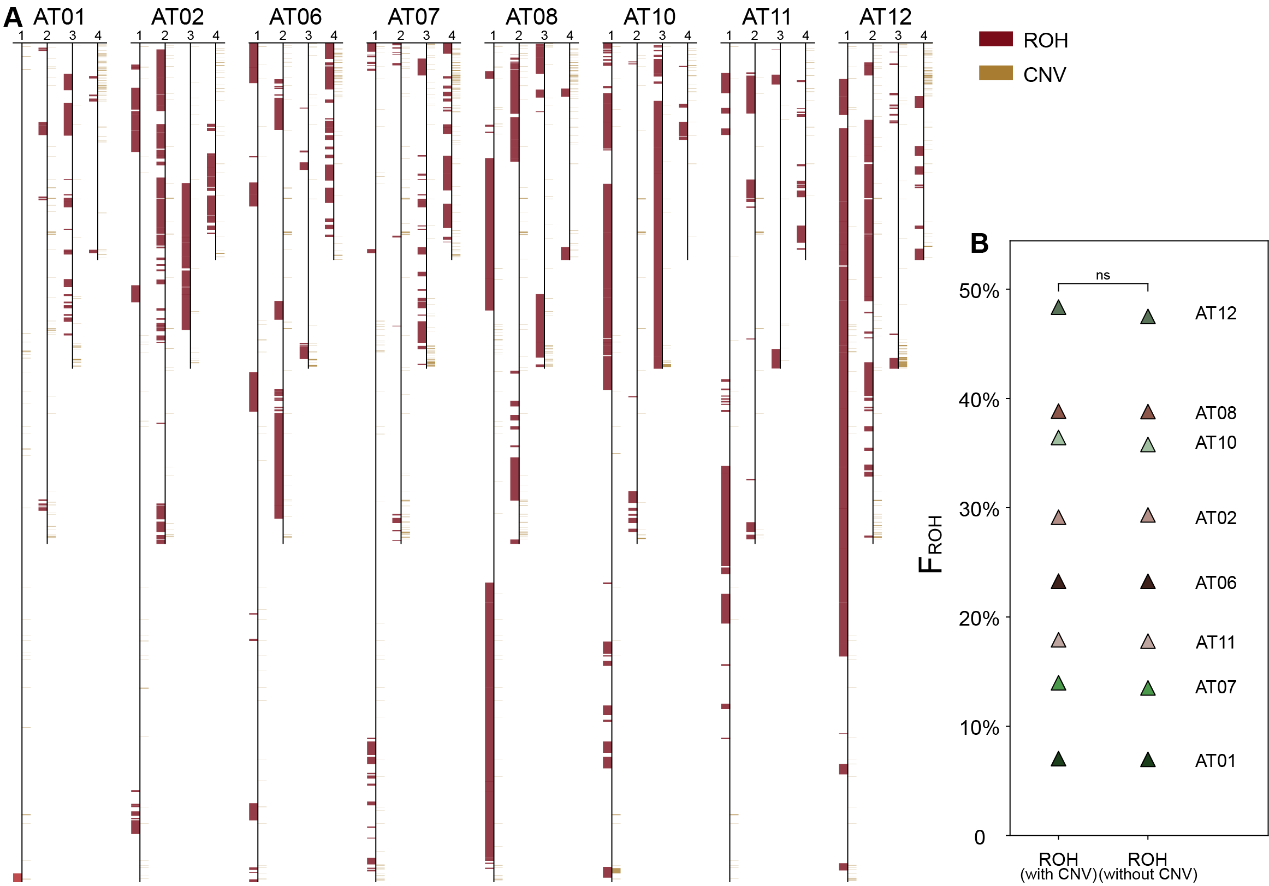


Figure S2. Effects of CNV regions on ROH detection. (A) Distribution of ROH and CNV on the genome. Each individual in the figure is arranged from left to right as chr1-chr4. (B) Original F_ROH_ and corrected F_ROH_ after removing ROH segments in CNV regions


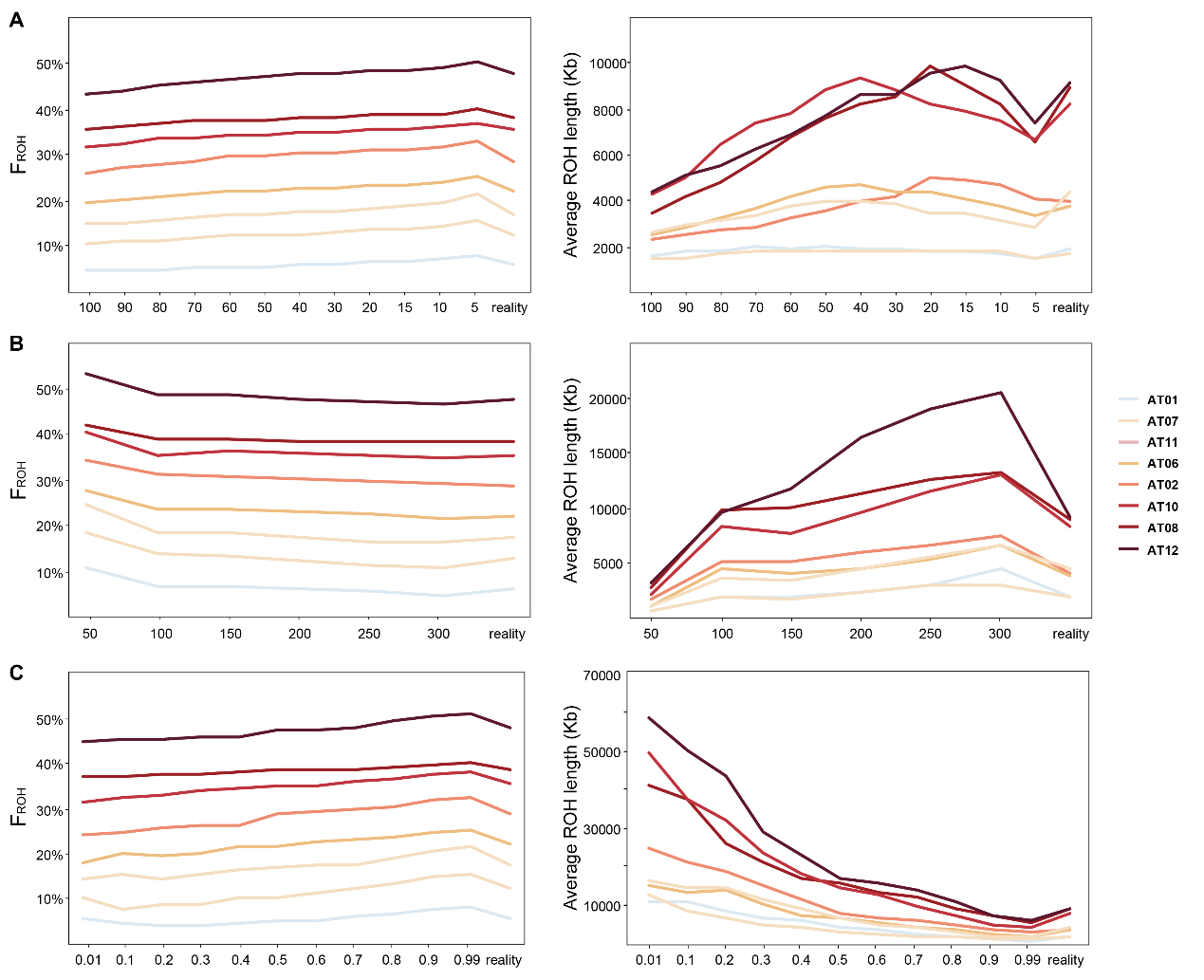


Figure S3. The detected F_ROH_ and average ROH length with the change of PLINK parameters under a 5× WGR data. A. Parameter "--homozyg-window-snp". B. Parameter "--homozyg-snp". C. Parameter "--indep-pairwise".


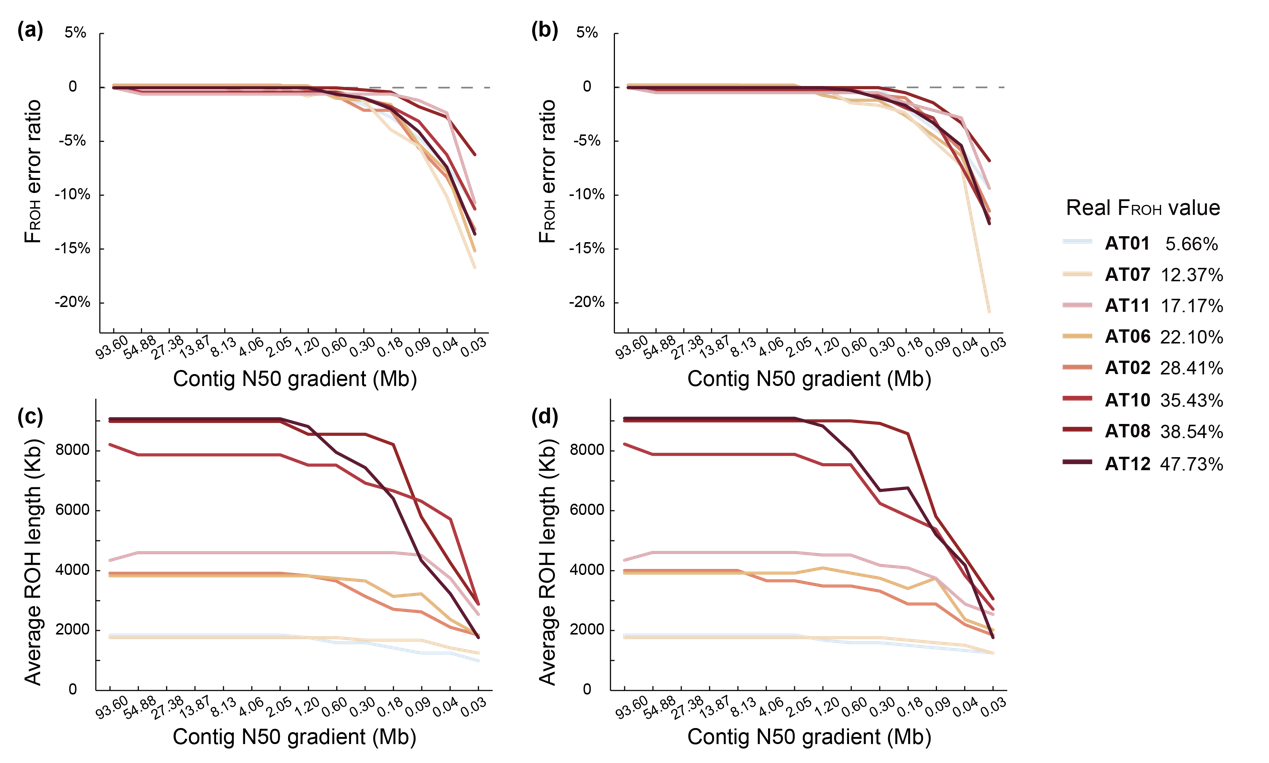


Figure S4. The impact of read length on the detection of ROH segments across the genome. (A) and (B) The ratio of detected F_ROH_ to the real F_ROH_ based on 100bp (A) and 150bp (B) simulated resequencing data with reference genomes of different continuities. (C) and (D) The average length of detected ROH segments based on 100bp (C) and 150bp (D) simulated resequencing data with reference genomes of different continuities.


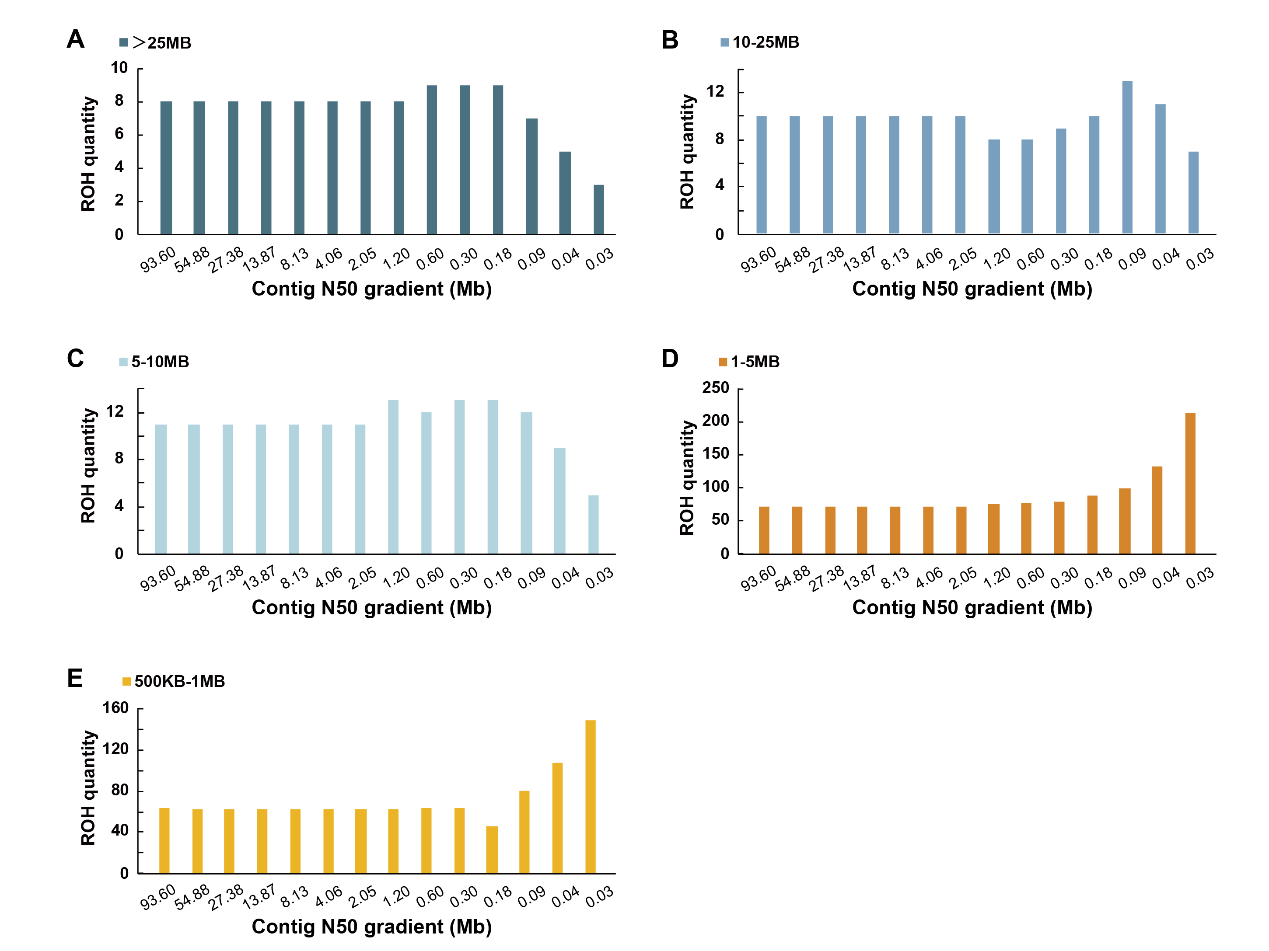


Figure S5. The changing trend of the number of ROH in different length intervals with genome continuity.


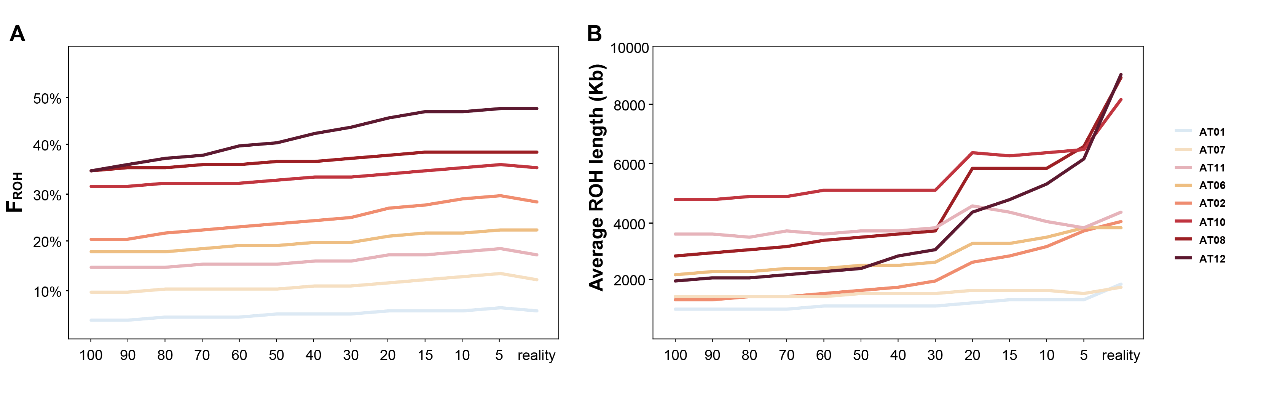


Figure S6. The detected F_ROH_ and average ROH length with the change of PLINK parameters of "*--homozyg-window-snp*" from 100 to 5, based on a reference genome with the contig N50 of 0.09Mb.


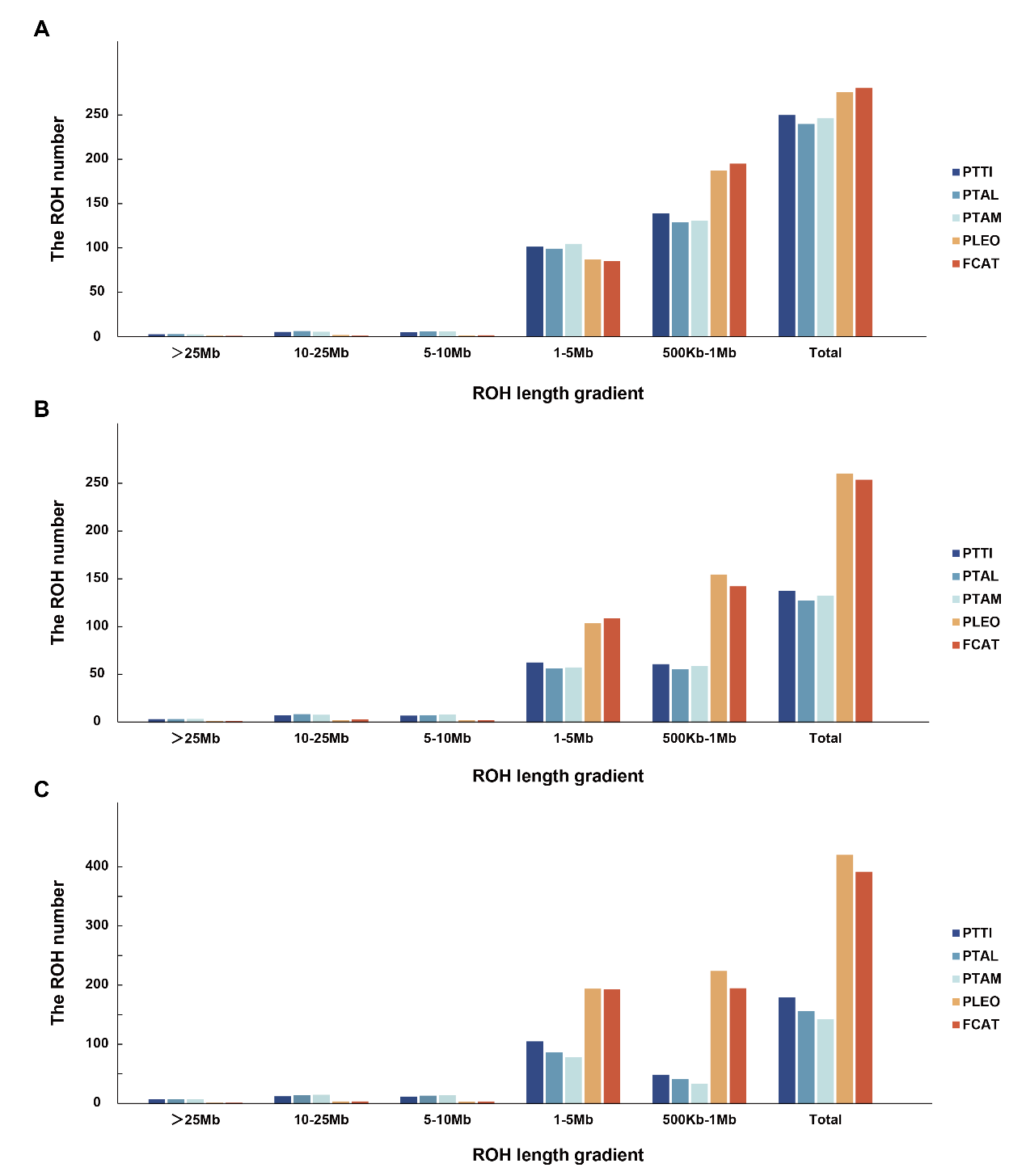


Figure S7. Comparison of the ROH detection with different reference genomes in the Bengal tiger (A), Amur tiger (B) and South China tiger (C) populations.

Figure S8. The change trend of the F_ROH_ and average ROH length after adjusting the parameters is shown in the group that used the lion reference genome (PLEO) to analyze the Bengal tiger population (BT). Here, the results of the Bengal tiger genome (PTTI) analysis are used as the true value for comparison.

**Supplementary Tables**

(These supplementary tables are saved in the *Supplementary Forms.docx* document.)

Table S1. The ideal reference genome for simulation.

Table S2. Simulated reference genome continuity gradient.

Table S3. Raw sequencing data from three tiger populations.

Table S4. Reference genome information for different tiger subspecies.

Table S5. The start and end positions of the true value of the ROH segments.

Table S6. ROH parameters: Group 1: The most complete genome, read length 100bp, different sequencing data amounts.

Table S7. ROH detection results of 5x sequencing data when adjusting the parameters.

Table S8. ROH parameters: Group 2: Genomes of different Contig N50, read length 100bp, 20× sequencing data volume.

Table S9.ROH parameters: Group 3: Genomes of different Contig N50, read length 150bp, 20× sequencing data volume.

Table S10. ROH detection results of sequencing data with five read lengths (all with a depth of 20×) on three reference genomes with different continuity.

Table S11. ROH detection results of reference genome Contig N50 of 0.09Mb when adjusting the parameters.

Table S12. F_ROH_ values obtained by comparing different reference genes with different tiger subspecies.

Table S13. ROH length distribution obtained by comparing different reference genes of different tiger subspecies.

Table S14. The changing trend of ROH detection, when analyzing Bengal tigers (BT) with lions (PLEO) as the reference genome, parameters were adjusted.
